# Transient Musical Hallucinations in a Young Adult Male Associated with Alcohol Withdrawal

**DOI:** 10.1155/2019/6546451

**Published:** 2019-11-03

**Authors:** Graham Blackman, Mao Fong Lim, Fahmida Mannan, Anthony David

**Affiliations:** ^1^Department of Psychosis Studies, Institute of Psychiatry, Psychology and Neuroscience, 16 De Crespigny Park, Camberwell, London, UK; ^2^GKT School of Medical Education, King's College London, UK; ^3^UCL Institute of Mental Health, University College London, London, UK

## Abstract

We present the case of a 25-year-old male who presented to A&E with isolated musical hallucinations, in the absence of audiological or neurological disease. The patient had a history of recreational drug use and a family history of psychosis. Hallucinations, which were preceded by discontinuation of alcohol and reinitiation of citalopram for depression, resolved spontaneously after three days. Aetiological factors are discussed alongside the existing literature. Whilst the underlying mechanisms underpinning musical hallucinations remain elusive, the case illustrates the potential role of alcohol withdrawal, serotonin toxicity, recreational drug use, and genetic vulnerability.

## 1. Introduction

Musical hallucinations (MH) are a form of complex auditory hallucinations whereby an individual experiences an instrumental and/or vocal melody in the absence of auditory stimuli [1]. MH were first described over a century ago, by the French psychiatrist Baillarger [2], and are estimated to occur in 0.16% of patients admitted to psychiatric hospitals [3]. They are more common in females and the elderly [1, 4] and are associated with a range of otological, neurological, and psychiatric disorders. Among organic disorders, hypoacusis, focal brain lesions, epilepsy, and drug toxicity/withdrawal states are most strongly associated with MH [1, 4–6]. Among psychiatric disorders, they are most often related to depression, bipolar, schizophrenia and schizoaffective disorder [1, 5, 6].

Despite the identification of several risk factors, the underlying pathophysiology underpinning MH remains, unclear. Converging evidence suggests activation of the auditory association cortex, and in particular the superior temporal sulcus, is associated with MH; other implicated regions include the orbitofrontal cortex, precuneus and basal ganglia [7]. When MH are secondary to an organic disorder, treatment is typically focused around the underlying cause. In cases of idiopathic MH, antidepressants, antiepileptics, acetylcholinesterase inhibitors, and antipsychotics have been used; however, the evidence base is limited [6].

We present a case of transient MH occurring in a young adult male, in the absence of audiological or neurological comorbidities, in whom multiple factors, including alcohol withdrawal, had a putative aetiological role. The case raises the possibility that onset of MH requires a necessary threshold which can be attained through one, or more contributory factors.

## 2. Case Report

A 25-year-old single unemployed white male, who lived with his parents, was awoken by a song by the psychedelic rock band ‘Animal Collective' playing continuously. The music, which consisted of instrumental and vocal components, appeared to come from outside his bedroom and was of equal loudness to the ambient surrounding. He heard the music in both ears in the absence of tinnitus, or any other otological symptoms. The song, which the patient had last listened to one month prior, had personal significance and initially evoked a positive emotional response.

**Figure 1:**
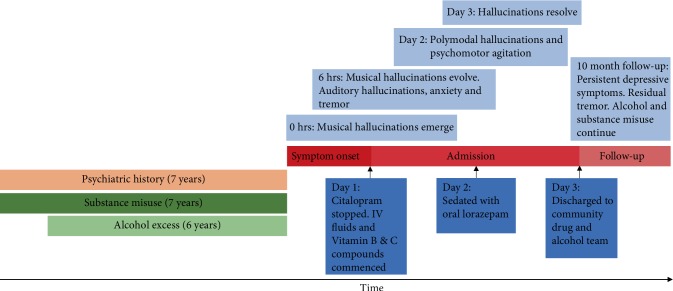
Timeline of events of musical hallucinations (MH). Dark blue text boxes indicate medical interventions over the course of admission. Timeline not to scale.

Six hours later, elements of the music began to fluctuate, such as the source location, content (songs began to merge, forming novel compositions), and controllability (the patient became able to control the music's characteristics). He also experienced a sudden onset of paroxysmal anxiety, tremor, and palpitations. Approximately one day later, he presented to A&E after becoming distressed by multiple unidentified voices talking to him in a derogatory manner, which were able to access and comment on his thoughts (see Figure 1 for timeline).

The patient did not have a history of neurological or audiological disease. He had a psychiatric history of recurrent depression since the age of nineteen, describing his mood as persistently low, with periods of deterioration without clear precipitants. He had trialled several anti-depressants with no significant improvement and had restarted Citalopram 40 mg ten days prior to the onset of MH. His mother had suffered from chronic psychosis for several years, requiring antipsychotic medication and several psychiatric admissions.

He reported a six-year history of excessive alcohol use. Over the preceding two years he consumed on average 80 units of alcohol per week, and described tremor, anxiety and paraesthesia upon abrupt cessation. He last consumed alcohol two days prior to symptom onset. He had a seven-year history of regular cannabis use, including high potency cannabis (“skunk”), which he had last used two weeks prior. He had also infrequently used the stimulant 3,4-methylenedioxymethamphetamine (MDMA), which he had last used one month prior and had experimented with the hallucinogens lysergic acid diethylamide (LSD), N,N-dimethyltryptamine (DMT) and Ketamine over a year prior.

## 3. Physical Examination

Examination on arrival to A&E revealed bilateral resting tremor of the upper limbs. There were occasional word finding difficulties, with no evidence of dysarthria or dysphasia. His mood was anxious and dysphoric, with reactive affect. There was no evidence of thought disorder, catatonia or delusional ideation. He experienced musical hallucinations in extracorporeal space, alongside second and third-person auditory hallucinations. Cognitively he was alert, however occasionally distracted by internal stimuli. Mini-mental state examination was normal (29/30) and he was fully orientated. He retained insight regarding his perceptual abnormalities. Neurological examination revealed symmetrical mydriasis (pupillary diameter 7–8 mm) which were equal and reactive to light. Cranial nerve examination was otherwise unremarkable, with no evidence of ophthalmoplegia or hypoacusis on bedside examination. Peripheral nerve examination revealed normal power, sensation, reflexes, and tone and no evidence of clonus. There was evidence of ataxia, including dysmetria on finger-nose examination, dysdiadochokinesis and instability on tandem gait.

## 4. Diagnostic Assessment

On arrival to A&E the patient was tachycardic (115 bpm) and hypertensive (162/88 mmHg) with a normal temperature (36.5 degrees). An ECG revealed sinus tachycardia and initial blood tests revealed normal renal and liver function, except for a raised gamma-glutamyl transferase (57 mmol/L) and mildly elevated bilirubin (23 mmol/L). C-reactive protein (CRP) and white cell count were in normal range and a venous blood gas revealed a slightly elevated lactate (1.2 mmol/L). Urine toxicology was negative for illicit substances. Electrolytes, including calcium and magnesium were normal, except for a mildly low phosphate (0.76 mmol/L). Creatine kinase was elevated upon admission (260 IU/L), however, normalised by the following day.

Alcoholic hallucinosis was considered, in view of chronic harmful use and recent discontinuation, along with serotonin toxicity considering the recent recommencement of citalopram. Also included in the differential list was a drug induced-psychosis and a nonorganic “functional” mental disorder. Finally, an organic cause, such as temporal lobe epilepsy was considered.

## 5. Interventions and Progress

The patient was admitted to a medical ward, with input by a psychiatric liaison service. Citalopram was discontinued and the patient was commenced on intravenous fluids and high potency vitamin B and C compounds for possible alcohol withdrawal. Antipsychotic medication was not initiated.

One day following admission, he developed psychomotor agitation, visual and tactile hallucinations and described “ghosts” passing through him. He continued to hear musical and second person auditory hallucinations. Insight was severely impaired during this period, and the patient required oral sedation with lorazepam due to the degree of agitation with good effect. After a further day, his hallucinatory and delusional symptoms fully-resolved, and he was discharged to a community drug and alcohol team.

## 6. Follow-Up and Outcomes

Ten months following discharge, the patient remained free of psychotic symptoms, however his mood remained dysthymic. He had been commenced on mirtazapine 15 mg daily with minimal improvement. Neurological examination revealed resting tremor of the upper limbs, dysmetria, and instability on tandem gait remained; however, dysdiadochokinesis had resolved. The patient continued to consume alcohol regularly (approximately 80 units per week), whilst recreational drug use was limited to occasional MDMA and ketamine consumption.

## 7. Discussion

The case describes an abrupt onset of transient MH in a young male, with subsequent visual, tactile, and auditory verbal hallucinations. Treatment was supportive and his psychotic symptoms resolved after 3 days.

Arguably the most convincing aetiological factor for the emergence of MH in this case was an alcohol withdrawal syndrome. The patient fulfilled the DSM-5 criteria for alcohol withdrawal through the presence of anxiety, psychomotor agitation, transient hallucinations, tremor, and autonomic dysfunction features [8]. This was further supported by symptoms coinciding with the typical peak window for alcohol withdrawal syndrome and improving with benzodiazepines. Furthermore, the development of other hallucinatory experiences commonly associated with alcohol withdrawal, including visual and tactile hallucinations are consistent with this hypothesis. Supporting the association between MH and alcohol withdrawal, a case series of 70 patients admitted for alcohol detoxification (with a history of alcohol-related hallucinations) revealed 63% had experienced musical hallucinations at some stage, suggesting the phenomena is closely associated with alcohol withdrawal [9].

A nonorganic “functional” mental disorder could also account for the development of MH. A history of recurrent depression and a first-degree relative with chronic psychosis—suggesting a genetic susceptibility toward psychosis—would support this hypothesis, with both disorders associated with MH [6]. However, the abrupt onset of symptoms in the absence of any prominent affective, or psychotic symptoms at the time would be atypical.

An aetiological role for citalopram, a selective serotonin reuptake inhibitor (SSRI), is also plausible, however less clear. Recommencing citalopram at 40 mg (the maximum licenced dose in the UK) without titration may have increased the likeliness of serotonin toxicity. Furthermore, the onset of symptoms coincided with predicted peak serum concentrations [10] and resolved after citalopram was discontinued. Symptoms consistent with serotonin toxicity include incoordination, tremor, tachycardia, mydriasis and agitation [11]. However, these could also be explained by an alcohol withdrawal syndrome and other features of serotonin toxicity with greater discriminant value, such as clonus, diaphoresis, and hyperreflexia [12] were absent. Intriguingly, there is contradictory evidence for the role of antidepressant medication, such as citalopram, in MH. In the largest case series of MH to date, consisting of 393 patients [1], four patients (1%) experienced MH in association with antidepressant use. However, there is also evidence suggesting antidepressants may have a potential therapeutic role in MH, particularly among the elderly [13].

The patient's use of recreational drugs with known psychotogenic properties, such as cannabis and ketamine, raises the possibility of substance misuse playing an aetiological factor. Auditory hallucinations are a common symptom of drug-induced psychosis [14] and there is evidence that this can include MH, particularly in association with Ketamine use [15]. However, to what extent these psychoactive substances could lead to MH, in the context of a period of abstinence is unclear.

Finally, a structural lesion or neurological disorder, such as temporal lobe epilepsy, cannot be excluded (in the absence of neuroimaging and neurophysiological investigation), however, can be considered unlikely in view of the transient and monophasic nature of the symptoms.

Arguably, a multifactorial explanation accounts for the emergence of MH most satisfactorily. Each factor alone may not have been enough, however the cumulative effects of two or more may have been sufficient to lead to the development of this intriguing mental phenomenon. For example, whilst an alcohol withdrawal syndrome may have been a major precipitating factor, additional clinical variables, such as genetic liability, may have contributed towards meeting a necessary threshold. This multifactorial hypothesis is supported by previous studies in MH suggesting that more than one aetiological factor is often present at the time of symptom onset [1]. Future research is indicated to disentangle contributory aetiological factors and their relative importance in MH.

The case provides a valuable contribution towards the possible pathophysiological mechanisms underpinning MH. Several theories have been proposed [5]. One hypothesis is that MH are due to reduced sensory input leading to a “perceptual release” phenomenon, akin to an auditory equivalent of Charles Bonnet syndrome [4], which is consistent with the finding that MH are more commonly observed in elderly patients with hearing loss. An alternative is that MH arises from “parasitic” musical memories which become inappropriately activated [5]. A Bayesian-based model, based on neurophysiological and phenomenological observations, proposes that musical hallucinations arise from top-down and bottom-up prediction errors [16]. Finally, it has been hypothesised that MH arise from “cerebral irritation” triggering activation of musical memories stored in neural networks, likely involving the auditory association cortex. The case lends particular support to this theory in view of the known excitotoxic effects of an alcohol withdrawal state [17]. Of relevance was the evidence of persistent cerebellar ataxia, which may be explained by alcohol-related cerebellar degeneration. Aberrant glutamatergic transmission is a putative mechanism for cerebellar damage in alcohol withdrawal syndromes [18], and is also thought to play an important role in psychotic disorders [19], suggesting that—whilst affecting different brain regions—there may be shared pathophysiological mechanisms underlying both neuropsychiatric phenomena.

Finally, it is noteworthy that musical hallucinations preceded all other hallucinatory phenomena by approximately one day. What may we infer from this? A review of the literature does not indicate whether the chronology of events in this case is typical for polymodal hallucinations, which include MH. However, it could be tentatively speculated that the superior temporal sulcus, or other brain regions implicated in the neural network underpinning musical hallucinations may be particularly sensitive to the effects of alcohol withdrawal, or other causative factors. Future observational research describing the sequence of psychopathology would be well placed to explore this intriguing question.

It is important to acknowledge the limitations of this study, namely that as a case report it has very limited generalizability. Furthermore, the absence of neuroimaging and neurophysiology make it difficult to definitively exclude an underlying neurological disorder. A strength, meanwhile, includes the detailed phenomenology described in temporal sequence.

Musical hallucinations are an important and underinvestigated area of psychiatry. Whilst several aetiological factors have been identified, the pathophysiological mechanisms are still not well understood. There is need for the systematic study of MH, including putative aetiological factors, to widen our understanding of this fascinating phenomenon and identify potential new treatment targets.
